# Intra-positional technical performance under different physical demands in elite football: a comparison between most demanding passage and extremely demanding passage

**DOI:** 10.3389/fphys.2026.1900931

**Published:** 2026-08-06

**Authors:** Zunqi Niu, Zhaoyang Wang, Yuntao Wang, Iyán Iván-Baragaño

**Affiliations:** 1 Hubei Provincial Research Center for Excellence Campus Football, Wuhan Sports University, Wuhan, China; 2 School of Physical Education and Sports Science, South China Normal University, Guangzhou, China; 3 Faculty of Physical Activity and Sports Sciences, University of Leon, Leon, Spain

**Keywords:** Chinese Super League, extremely demanding passage, football, most demanding passage, performance analysis

## Abstract

**Introduction:**

This study investigated positional differences in technical performance between the Most Demanding Passage (MDP) and the Extremely Demanding Passage (EDP) in elite football. EDP was defined as the top 5% of match phases based on position-specific high-intensity running (HIR) distributions.

**Methods:**

A total of 4,102 outfield player observations from 176 Chinese Super League matches were analysed, including 3,664 MDP and 438 EDP observations. Technical indicators were compared between MDP and EDP using both bivariate statistical analysis and a Random Forest classifier combined with SHAP (SHapley Additive exPlanations) to identify the most influential variables contributing to differences between conditions.

**Results:**

Significant intra-positional differences were found. Central defenders increased short-pass accuracy (p < .001, ES = 0.62) and ball-carrying distance (p = .01, ES = 0.52), while reducing long passes (p < .001, ES = –0.44). Full-backs exhibited lower long-pass volume (p < .001, ES = 0.57) and forward pass length (p < .001, ES = 0.48). Central midfielders recorded fewer successful long passes (p < .001, ES = 0.34) and more ball losses (p = .03, ES = 0.30). Forwards showed declines in lateral passing (p = .02, ES = 0.37) and individual possession time (p = .002, ES = 0.36). Wide midfielders exhibited minimal changes between conditions. The Random Forest and SHAP analysis further indicated that passing actions (e.g., short passes, long passes) and ball progression behaviours (e.g., ball carrying) were the primary contributors to distinguishing EDP from MDP across playing positions.

**Discussion:**

Overall, these results suggest that technical performance varies in a position-dependent manner under extreme physical demands, highlighting the importance of role-specific training strategies that replicate high-intensity match conditions.

## 1 Introduction

Modern football necessitates that players keep high levels of physical and technical performance during intense time pressure and fatigue (Barthelemy et al., 2024). For improved an understanding of peak match demands, researchers have introduced the concept of the Most Demanding Passage (MDP) (Martín-García et al., 2018), which generally indicates the period during which a player covers the most high-intensity running (HIR) distance (≥19.8 km/h) (Novak et al., 2021), usually determined using rolling averages across different time windows (Fereday et al., 2020). The MDP has become recognized as a frequent criterion for match intensity and has informed training and recovery protocols in elite football (Martín-García et al., 2018).

However, MDPs show substantial variation related to positional demands, individual physical characteristics, and match-specific tactical factors (Martín-Fuentes et al., 2021; Martín-García et al., 2018; Piñero et al., 2023). This variability occurs not only between players in different roles within a single game but also across matches for the same player (Bush et al., 2015; Lobo-Triviño et al., 2025). Thoseby et al. (2023) reported coefficients of variation for high-speed running distance ranging from 20.6% to 29.8% across matches, indicating that peak physical efforts fluctuate considerably depending on the context. As a result, the MDP identified in one match may not reliably match with the extreme physical demands reported in another. This inconsistency limits the comparability of MDP values between players and contexts, limiting the validity of it as a reliable indicator of maximum exertion. Although MDPs reflect the most physically demanding segments of individual matches (Lino-Mesquita et al., 2025), they may not accurately reflect the highest level of exertion that characterizes a player’s highest physical demands. These factors highlight the necessity for an improved approach that considers inter-positional and inter-match variability in identifying peak physical demand.

However, recent evidence indicates that MDPs may not always capture players’ true peak physical demands. To address this limitation, Niu et al. (2025) introduced the concept of the Extremely Demanding Passage (EDP), which identifies match phases in which players exceed position-specific thresholds derived from the distribution of high-intensity running across multiple matches and playing positions. EDP is derived from MDP observations and identifies the most extreme phases within the MDP distribution by applying position-specific thresholds. The EDP differs from the MDP, which is based on peak periods within a single match. In contrast, the EDP is constructed from repeated observations across matches and positions, allowing it to capture the upper tail of physical demand distributions. This framework therefore reflects recurrent exposure to extreme-intensity conditions rather than isolated within-match peaks. As a result, it reduces sensitivity to single-match fluctuations and provides a more stable representation of maximal physical demands in elite football. Compared to traditional MDPs, EDPs have been shown to involve significantly higher physical loads, including increased total distance (+18.6%), sprint distance (+58.5%), and sprint efforts (+61.4%), thereby highlighting a distinct level of intensity.

Extreme physical exertion in football, as represented by EDP, frequently results in fatigue-related limitations in motor coordination, decision-making, and technical execution (Angius et al., 2022; Dambroz et al., 2022). Empirical evidence from intermittent high-intensity exercise and match-play studies has consistently demonstrated that fatigue negatively affects passing accuracy, ball control, and technical execution efficiency (Mohr et al., 2005; Rampinini et al., 2009). Furthermore, such impairments are particularly evident during repeated high-intensity phases and transitional periods of match play, where both physical and cognitive demands are simultaneously elevated (Bangsbo et al., 2006). In modern football, where short bursts of high-intensity pressing and rapid transitions occur frequently, these fatigue-induced constraints are likely to have position-specific impacts on technical performance.

Although considerable study has focused on the physical demands of competition, there has been limited examination of how technical performance is influenced when players perform under near-maximal physical stress. The subject is becoming more important in modern football, where high-intensity pressing and rapid transitions produce short but intense phases of effort (Andrienko et al., 2017; Brîndescu et al., 2021). Given that physical load patterns differ by position (Teixeira et al., 2024), the response of technical execution to differences in physical intensity may also vary across each playing position. Understanding these intra-positional variations is crucial for recognizing how technical performance differs across different levels of physical demand.

This study aims to compare technical performance between two levels of match intensity, EDP and MDP, for each of the five outfield positions: central defenders (CD), full-backs (FB), central midfielders (CM), wide midfielders (WM), and forwards (FW). It is hypothesized that technical performance differs between EDP and MDP conditions across playing positions due to differences in positional physical and technical demands. Analyzing intra-positional differences in technical performance under extreme physical demand may provide important insights into the effect of intense exertion on technical output and guide the development of position-specific training strategies.

## 2 Materials and method

### 2.1 Participants

A total of 4,102 observations were included in this study, including 3,664 MDP observations and 438 EDP observations (CD: 975 observations, 861 MDP observations, 114 EDP observations; FB: 959 observations, 882 MDP observations, 77 EDP observations; CM: 980 observations, 868 MDP observations, 112 EDP observations; WM: 618 observations, 565 MDP observations, 53 EDP observations; FW: 570 observations, 488 MDP observations,82 EDP observations), corresponding to 176 matches in the 2021 Chinese Super League (CSL) season. To mitigate potential biases, substitute players were excluded from this research.

The local university’s ethics committee granted approval for this study (SCNU-SPT-2025-008). Given the retrospective and anonymized nature of the data, the Ethics Committee of South China Normal University approved the waiver of written informed consent from participants.

### 2.2 Procedure

Running and technical data for all players were recorded using a semi-automated multi-camera tracking system (STATS Pro^®^, Sport-Universal Process, Nice, France), making up eight stable, coordinated cameras set on the stadium, working at a sampling frequency of 25 Hz during the matches (Castellano et al., 2014). The signals and angles received from the sensors were systematically transformed into digital data and saved on a computer for later study. All data was recovered post-match utilizing the provided private software (STATS Viewer, version 3.6.0.3). This technology’s effectiveness and reliability have been previously validated (Zubillaga et al., 2007).

### 2.3 Identification of MDP and EDP

In accordance with prior studies, high-intensity running (HIR) was defined as movement at speeds equal to or exceeding 19.8 km/h (Fereday et al., 2020; Oliva-Lozano et al., 2020). The MDP for each player was identified by applying a 5-minute rolling average to detect the period with the maximum distance covered at high-intensity running speeds (≥19.8 km/h).

Thereafter, the mean and standard deviation (SD) of the MDP values were determined independently for each playing position (CD, FB, CM, WM, and FW, respectively). Similar with a prevalent machine learning-based outlier detection approach (Li et al., 2022), the EDP was characterized as any MDP value that beyond the positional mean by more than 1.65 standard deviations (i.e., M + 1.65SD) (Niu et al., 2025). This method retained only the top 5% of the most physically demanding phases for the next analysis, therefore capturing the most extreme instances of exertion during elite match play. This approach is consistent with percentile-based methods (e.g., 90th percentiles) commonly used in the match analysis literature. The selection of this threshold was based on the statistical property of the normal distribution, where values above 1.65 SD from the mean correspond approximately to the upper 5% of observations. This upper tail region is commonly considered to represent extreme values within a distribution and was therefore used to identify the most exceptional MDP phases (Tukey, 1977). Alternative approaches such as interquartile range (IQR)-based thresholds or fixed percentile cut-offs may also be applied depending on the analytical objective. However, the present study adopted the 1.65 SD criterion to ensure consistency with prior outlier detection frameworks and to maintain comparability across positional distributions.

### 2.4 Variables

In line with previous studies on MDP analysis (Fang et al., 2025), 28 technical performance-related variables were selected to examine the differences in technical performance between the EDP and MDP phases. The operational definitions of these variables are presented in **Table 1**.

**Table 1:** Technical performance-related variables.

Group	Variable	Operational definition
Technical performance	50challenge	Duels where both players contest the ball equally
	AvgTmIndPos	Average time in each individual ball possession
	BallTch	The total number of ball touches
	S.BallTch	Total successful ball touches
	BallLost	Total number of times possession was lost
	BallWon	Total number of times possession was won
	N. Clearance	Total number of times the ball was cleared
	AvgBallTch	Average ball touches per individual possession
	N.TchIndPoss	Total ball touches in individual possession
	TotIndPoss	The total number of individual possessions
	TotTmIndPos	Total time in individual possession
	N.LateralPass	The total number of lateral passes
	S.LateralPass	The total number of successful lateral passes
	N.LongPass	Total long passes (>25 meters) attempted
	S.LongPass	Total successful long passes
	N.FwdPass	Total forward passes made toward the opponent’s half
	N.Pass	Total passes attempted by a player
	S.Pass	Total successful passes made by a player
	RunBallAvgDur	Average time a player spends running with the ball
	RunBallAvgLen	Average distance of each effort running with the ball
	N.ShortPass	Total short passes (<15 meters)
	S.ShortPass	Total successful short passes
	Shot	Total attempts to score a goal
	N.Tackle	Total tackles attempted to win the ball
	S.Tackle	Total successful tackles made
	AvgFwdLen	Average distance of forward passes
	GainPosse	Total times a player gains possession of the ball
	N.Distrib	Total number of ball distributions

### 2.5 *A priori* sample size estimation and justification

A conventional *a priori* sample size calculation was not performed because this study used a retrospective observational design based on all eligible observations obtained from the 2021 Chinese Super League season rather than prospectively recruiting participants. Thus, the sample size was determined by data availability and the predefined inclusion criteria. Specifically, all observations derived from 176 matches were screened, and only eligible MDP and EDP observations from non-substitute players were retained for analysis. This resulted in a final sample of 4,102 observations, including 3,664 MDP observations and 438 EDP observations. Given the use of an exhaustive season-based dataset, the present study prioritized complete inclusion of all eligible observations over recruitment-based sample size targeting. In addition, the repeated-measures structure of the data was accounted for in the statistical models, which further supported the appropriateness of the available sample for the planned analyses.

### 2.6 Data analysis

A comparative analysis was carried out for each of the specific positions analyzed, in order to test the null hypothesis of mean differences for each of the variables analyzed based on MDP and EDP. Normality and homoscedasticity for each variable and group were tested using the Kolmogorov-Smirnov and Levene’s tests, respectively, with a significance level of p <.05. Differences were assessed using the Student’s t-test for independent samples for normally distributed variables, and the Mann-Whitney U test for non-normally distributed variables. Effect size was calculated only for those variables in which statistically significant differences were observed, using Cohen’s d, and categorized as trivial (ES <.20), small (ES <.60), or moderate (ES < 1.20) (Batterham and Hopkins, 2006). Each observation represented a match-phase event extracted from competitive matches, and analyses were performed at the population level to describe performance differences under different intensity conditions.

Variables showing significant differences in the bivariate analysis were selected for the multivariate model. This step was used to reduce the number of variables and focus on indicators that showed clear differences between EDP and MDP. The Random Forest model was then applied to further explore multivariate patterns across technical variables. This second step complements the statistical analysis by capturing nonlinear relationships and interactions between variables. A Random Forest (RF) classifier was developed utilizing Python (v3.11.8), with scikit-learn (v1.3.0), imbalanced-learn (v0.12.0), and SHAP (v0.45.0) libraries. To implement the RF classifier, a grid search method was applied to optimize key hyperparameters, including the number of estimators (n_estimators = 100 to 500), maximum tree depth (max_depth = 5 to 20), and the minimum number of samples required to split an internal node (min_samples_split = 2 to 10). The final hyperparameters were selected based on 5-fold cross-validation accuracy within the training dataset. The dataset was randomly divided into training (70%) and testing (30%) subsets, and class imbalance was decreased using the Synthetic Minority Over-sampling Technique (SMOTE).

The evaluation of model performance was conducted utilizing standard classification criteria, including precision, recall, and the F1-score. SHapley Additive exPlanations (SHAP) were utilized to explain the model’s outputs, and SHAP values were computed for each feature in the test set to provide both global (mean absolute SHAP values) and local (individual sample) interpretability. Visualization of SHAP values was performed through beeswarm plots and bar plots, with features ranked by average absolute SHAP contribution. Positive SHAP values indicated a greater likelihood of classification as EDP, while negative values reflected association with MDP.

In contrast compared to traditional statistical tests, the RF + SHAP framework provided a more robust and interpretable evaluation of feature importance by addressing non-linear interactions, multicollinearity, and imbalanced class distributions (Breiman, 2001; Lundberg and Lee, 2017). It complements the statistical tests by offering a different perspective on feature contribution across EDP and MDP conditions.

## 3 Results

**Table 2** shows that the models for CD, FB, CM, and FW exhibited robust discriminative accuracy and consistency. The WM model, however, failed to meet these criteria and was hence eliminated from further analysis.

**Table 2:** Model robustness and discriminative accuracy by playing position.

Position	Accuracy	Macro precision	Macro recall	Macro F1	Min F1	Std F1
CD	0.77	0.78	0.77	0.77	0.75	0.01
FB	0.73	0.74	0.73	0.73	0.7	0.02
CM	0.81	0.82	0.81	0.81	0.8	0.01
WM	0.55	0.52	0.56	0.46	0.08	0.24
FW	0.72	0.72	0.72	0.72	0.7	0.01

**Tables 3**–**7** presents the descriptive and bivariate results for the variables related to the technical performance of outfield players in the CSL during the EDP and MDP phases. **Figures 1**–**4** show the results of the multivariate analysis using SHAP summary plots for each playing position (CD, FB, CM, FW), highlighting how important each variable is and how they contribute in different ways. In the beeswarm plots, each dot represents an individual observation, with color indicating the feature value (red = high, blue = low). Positive SHAP values reflect a greater contribution to the classification of EDP (i.e., EDP is the positive class), while negative values indicate a stronger association with MDP. The variables are ranked in descending order of their mean absolute SHAP values, reflecting their relative importance in the classification model.

**Table 3:** Technical performance comparison between EDP and MDP for CD.

Variable	EDP (n=114)	MDP (n=861)	T	P	Cohen’s d
50challenge	0.34 ± 0.68	0.32 ± 0.58	0.19	0.85	
AvgTmIndPos	3.48 ± 2.81	3.17 ± 3.00	0.76	0.45	
BallTouch	9.19 ± 6.17	7.53 ± 5.63	2.17	0.03	0.29
S.BallTouch	8.42 ± 5.98	6.66 ± 5.56	2.35	0.01	0.32
BallsLost	0.61 ± 0.79	0.66 ± 0.84	-0.43	0.66	
BallsWon	0.75 ± 0.97	0.98 ± 1.00	-1.75	0.08	
N.Clearances	0.05 ± 0.22	0.12 ± 0.35	-2.23	0.02	-0.2
AvgBallTch	5.26 ± 3.10	4.74 ± 3.22	1.18	0.23	
N.TchIndPoss	8.44 ± 5.98	7.15 ± 5.51	1.74	0.08	
TotIndPoss	4.2 ± 2.5	3.5 ± 2.3	2.24	0.03	0.28
TotTmIndPos	5.88 ± 4.65	4.82 ± 4.74	1.66	0.09	
N.LateralPass	1.27 ± 1.48	1.66 ± 1.64	-1.77	0.07	
S.LateralPass	1.24 ± 1.48	1.57 ± 1.61	-1.54	0.12	
N.LongPass	0.66 ± 0.96	1.21 ± 1.25	-4.17	<.001	-0.44
S.LongPass	0.49 ± 0.83	0.97 ± 1.15	-4.14	<.001	-0.42
N.PassFwd	1.47 ± 1.23	1.13 ± 1.07	2.09	0.04	0.32
N.Passing	3.49 ± 2.19	3.10 ± 2.16	1.33	0.18	
S.Passing	3.22 ± 2.28	2.66 ± 2.09	1.99	0.047	0.27
RunBallAvgDur	1.30 ± 1.25	0.99 ± 1.19	1.89	0.06	
RunBallAvgLen	5.82 ± 7.04	3.36 ± 4.55	2.64	0.01	0.52
N.ShortPass	1.00 ± 0.91	0.52 ± 0.76	4.57	<.001	0.62
S.ShortPass	0.92 ± 0.93	0.43 ± 0.72	3.89	<.001	0.66
Shot	0.05 ± 0.22	0.02 ± 0.14	1.01	0.31	
N.Tackle	0.17 ± 0.37	0.15 ± 0.39	0.28	0.77	
S.Tackle	0.10 ± 0.30	0.12 ± 0.35	-0.41	0.68	
AvgFwdLen	26 ± 20	33 ± 26	-2.19	0.031	-0.24
GainPosse	0.69 ± 0.93	0.90 ± 0.97	-1.61	0.11	
N.Distrib	3.7 ± 2.4	4.9 ± 3.3	1.71	0.09	

**Table 4:** Technical performance comparison between EDP and MDP for FB.

Variable	EDP (n=77)	MDP (n=882)	T	P	Cohen’s d
50challenge	0.22 ± 0.53	0.24 ± 0.49	-0.19	0.84	
AvgTmIndPos	3.27 ± 3.49	3.50 ± 3.05	-0.46	0.64	
BallTouch	7.20 ± 5.61	8.65 ± 5.64	-1.6	0.11	
S.BallTouch	6.15 ± 5.23	7.45 ± 5.39	-1.51	0.13	
BallsLost	0.83 ± 0.86	0.91 ± 0.98	-0.51	0.61	
BallsWon	0.59 ± 0.67	0.75 ± 0.84	-1.23	0.21	
N.Clearances	0.02 ± 0.16	0.09 ± 0.29	-2.48	0.02	-0.23
AvgBallTch	5.25 ± 4.21	5.45 ± 3.30	-0.36	0.72	
N.TchIndPoss	6.66 ± 4.44	8.21 ± 5.42	-1.77	0.08	
TotIndPoss	4.21 ± 4.38	5.26 ± 4.42	-1.62	0.11	
TotTmIndPos	1.20 ± 0.78	1.38 ± 1.34	-1.46	0.14	
N.LateralPass	1.07 ± 0.79	1.38 ± 1.34	-1.38	0.17	
S.LateralPass	1.07 ± 0.79	1.21 ± 1.29	-1.05	0.29	
N.LongPass	0.22 ± 0.57	0.76 ± 0.98	-5.56	<.001	-0.57
S.LongPass	0.10 ± 0.30	0.55 ± 0.84	-7.74	<.001	-0.55
N.PassFwd	0.66 ± 0.73	1.00 ± 1.08	-1.97	0.049	-0.32
N.Passing	2.49 ± 1.34	3.29 ± 2.18	-3.54	<.001	-0.38
S.Passing	2.20 ± 1.32	2.77 ± 2.04	-2.57	0.01	-0.29
RunBallAvgDur	0.92 ± 1.04	1.21 ± 1.58	-1.15	0.25	
RunBallAvgLen	3.22 ± 4.46	3.98 ± 4.75	-1	0.31	
N.ShortPass	0.80 ± 0.78	0.90 ± 1.11	-0.53	0.59	
S.ShortPass	0.68 ± 0.76	0.76 ± 1.04	-0.47	0.64	
Shot	0.05 ± 0.21	0.2 ± 0.15	0.75	0.45	
N.Tackle	0.15 ± 0.42	0.17 ± 0.41	-0.32	0.75	
S.Tackle	0.05 ± 0.21	0.12 ± 0.34	-1.96	0.55	
AvgFwdLen	16.96 ± 16.08	28.01 ± 23.68	-4.11	<.001	-0.48
GainPosse	0.59 ± 0.67	0.70 ± 0.81	-0.86	0.38	
N.Distrib	2.88 ± 1.61	3.62 ± 2.30	-2.77	0.008	-0.33

**Table 5:** Technical performance comparison between EDP and MDP for CM.

Variable	EDP (n=112)	MDP (n=868)	T	P	Cohen’s d
50challenge	0.29 ± 0.49	0.28 ± 0.53	0.17	0.86	
AvgTmIndPos	3.33 ± 2.43	3.48 ± 3.35	-0.34	0.73	
BallTouch	8.04 ± 5.86	8.64 ± 6.23	-0.7	0.48	
S.BallTouch	6.62 ± 5.53	7.58 ± 6.04	-1.15	0.25	
BallsLost	1.15 ± 1.11	0.85 ± 0.95	2.17	0.03	0.3
BallsWon	1.00 ± 0.90	0.80 ± 0.94	1.58	0.12	
N.Clearances	0.05 ± 0.23	0.06 ± 0.25	0.16	0.88	
AvgBallTch	5.11 ± 3.11	5.61 ± 3.53	-1.02	0.31	
N.TchIndPoss	7.73 ± 5.92	8.23 ± 6.04	-0.59	0.55	
TotIndPoss	3.44 ± 2.26	3.78 ± 2.34	-1.04	0.29	
TotTmIndPos	4.93 ± 4.72	5.28 ± 5.68	-0.44	0.66	
N.LateralPass	1.64 ± 1.58	1.74 ± 1.58	-0.49	0.62	
S.LateralPass	1.36 ± 1.47	1.58 ± 1.54	-1.02	0.31	
N.LongPass	0.64 ± 0.78	0.99 ± 1.08	-2.35	0.019	-0.33
S.LongPass	0.45 ± 0.68	0.79 ± 0.98	-3.35	<.001	-0.34
N.PassFwd	0.71 ± 0.81	0.92 ± 0.99	-1.55	0.12	
N.Passing	2.84 ± 2.15	3.19 ± 2.20	-1.14	0.25	
S.Passing	2.13 ± 2.07	2.70 ± 2.11	-1.95	0.05	
RunBallAvgDur	1.18 ± 1.28	1.21 ± 1.99	-0.088	0.93	
RunBallAvgLen	4.52 ± 5.22	4.14 ± 4.65	0.58	0.56	
N.ShortPass	0.93 ± 1.06	0.83 ± 1.00	0.72	0.46	
S.ShortPass	0.62 ± 0.87	0.67 ± 0.92	-0.41	0.68	
Shot	0.05 ± 0.23	0.06 ± 0.26	-0.28	0.78	
N.Tackle	0.36 ± 0.49	0.18 ± 0.44	2.67	0.01	0.41
S.Tackle	0.24 ± 0.43	0.13 ± 0.36	1.85	0.06	
AvgFwdLen	22.52 ± 17.31	29.62 ± 24.85	-2.86	0.006	-0.29
GainPosse	0.91 ± 0.78	0.76 ± 0.90	1.41	0.16	
N.Distrib	3.00 ± 2.15	3.38 ± 2.26	-1.2	0.23	

**Table 6:** Technical performance comparison between EDP and MDP for WM.

Variable	EDP (n=53)	MDP (n=565)	T	P	Cohen’s d
50challenge	0.24 ± 0.56	0.29 ± 0.54	-0.37	0.71	
AvgTmIndPos	2.70 ± 3.22	3.77 ± 3.18	-1.36	0.17	
BallTouch	6.94 ± 7.32	8.65 ± 6.38	-1.08	0.28	
S.BallTouch	6.00 ± 6.65	7.35 ± 5.92	-0.92	0.36	
BallsLost	0.88 ± 1.16	1.05 ± 1.08	0.64	0.52	
BallsWon	1.00 ± 0.94	0.62 ± 0.83	0.78	0.06	
N.Clearances	0	0.05 ± 0.24	-5.63	<.001	-0.23
AvgBallTch	4.89 ± 4.24	5.92 ± 4.02	-1.03	0.30	
N.TchIndPoss	6.76 ± 7.02	8.27 ± 6.19	-0.98	0.32	
TotIndPoss	3.06 ± 2.01	3.47 ± 2.23	-0.74	0.46	
TotTmIndPos	3.95 ± 5.28	5.39 ± 4.93	-1.19	0.24	
N.LateralPass	1.18 ± 1.18	1.30 ± 1.29	-0.39	0.69	
S.LateralPass	1.0 ± 1.22	1.09 ± 1.21	-0.32	0.75	
N.LongPass	0.24 ± 0.44	0.59 ± 0.87	-3.14	0.005	-0.41
S.LongPass	0.24 ± 0.44	0.44 ± 0.76	-1.83	0.083	
N.PassFwd	0.65 ± 0.49	0.66 ± 0.91	-0.12	0.91	
N.Passing	2.47 ± 1.59	2.58 ± 1.95	-0.23	0.81	
S.Passing	2.18 ± 1.55	2.09 ± 1.79	0.21	0.84	
RunBallAvgDur	0.80 ± 1.11	1.16 ± 1.09	-1.35	0.18	
RunBallAvgLen	3.84 ± 6.11	4.30 ± 4.42	-0.42	0.67	
N.ShortPass	1.06 ± 0.83	0.83 ± 0.99	0.94	0.35	
S.ShortPass	0.88 ± 0.78	0.64 ± 0.88	1.11	0.27	
Shot	0	0.13 ± 0.36	-8.85	<.001	-0.38
N.Tackle	0.12 ± 0.33	0.15 ± 0.42	-0.36	0.72	
S.Tackle	0.12 ± 0.33	0.10 ± 0.35	0.21	0.83	
AvgFwdLen	16.25 ± 14.92	22.26 ± 23.76	-1.03	0.30	
GainPosse	0.94 ± 0.83	0.57 ± 0.77	1.92	0.05	
N.Distrib	2.53 ± 1.62	2.83 ± 2.07	-0.58	0.56	

**Table 7:** Technical performance comparison between EDP and MDP for FW.

Variable	EDP (n=82)	MDP (n=488)	T	P	Cohen’s d
50challenge	0.12 ± 0.39	0.44 ± 0.67	-4.85	<.001	-0.49
AvgTmIndPos	2.09 ± 1.96	2.97 ± 2.93	-2.7	0.009	-0.31
BallTouch	5.07 ± 3.68	6.40 ± 5.05	-2.21	0.03	-0.27
S.BallTouch	3.65 ± 3.69	6.40 ± 5.04	-2.22	0.02	-0.35
BallsLost	1.26 ± 1.21	0.98 ± 0.97	1.42	0.16	
BallsWon	0.49 ± 0.73	0.47 ± 0.72	0.21	0.83	
N.Clearances	0	0.03 ± 0.19	-4.08	<.001	-0.18
AvgBallTch	3.94 ± 2.65	4.79 ± 3.67	-1.95	0.05	
N.TchIndPoss	4.91 ± 3.57	6.09 ± 4.88	-2.02	0.04	-0.25
TotIndPoss	2.84 ± 1.68	2.74 ± 1.76	0.34	0.74	
TotTmIndPos	2.57 ± 2.53	3.92 ± 3.86	-3.2	0.002	-0.36
N.LateralPass	0.63 ± 0.98	1.02 ± 1.06	-2.31	0.02	-0.37
S.LateralPass	0.49 ± 0.88	0.81 ± 0.95	-2.16	0.03	-0.34
N.LongPass	0.28 ± 0.70	0.44 ± 0.74	-1.44	0.15	
S.LongPass	0.12 ± 0.62	0.32 ± 0.62	-2.07	0.04	-0.33
N.PassFwd	0.42 ± 0.66	0.40 ± 0.66	0.18	0.85	
N.Passing	1.79 ± 1.39	1.93 ± 1.54	-0.57	0.56	
S.Passing	1.42 ± 1.27	1.50 ± 1.36	-0.38	0.7	
RunBallAvgDur	0.46 ± 0.75	0.82 ± 0.92	-2.49	0.01	-0.4
RunBallAvgLen	1.48 ± 2.56	3.34 ± 4.18	-4.33	<.001	-0.45
N.ShortPass	0.84 ± 0.97	0.71 ± 0.91	0.89	0.37	
S.ShortPass	0.67 ± 0.92	0.52 ± 0.76	1.1	0.27	
Shot	0.28 ± 0.45	0.17 ± 0.39	1.59	0.06	
N.Tackle	0.05 ± 0.21	0.11 ± 0.34	-1.7	0.09	
S.Tackle	0.05 ± 0.21	0.06 ± 0.26	-0.42	0.68	
AvgFwdLen	14.68 ± 22.43	14.53 ± 19.44	0.048	0.96	
GainPosse	0.42 ± 0.63	0.43 ± 0.68	-0.1	0.92	
N.Distrib	1.84 ± 1.44	2.03 ± 1.61	-0.75	0.45	

**Figure 1:**
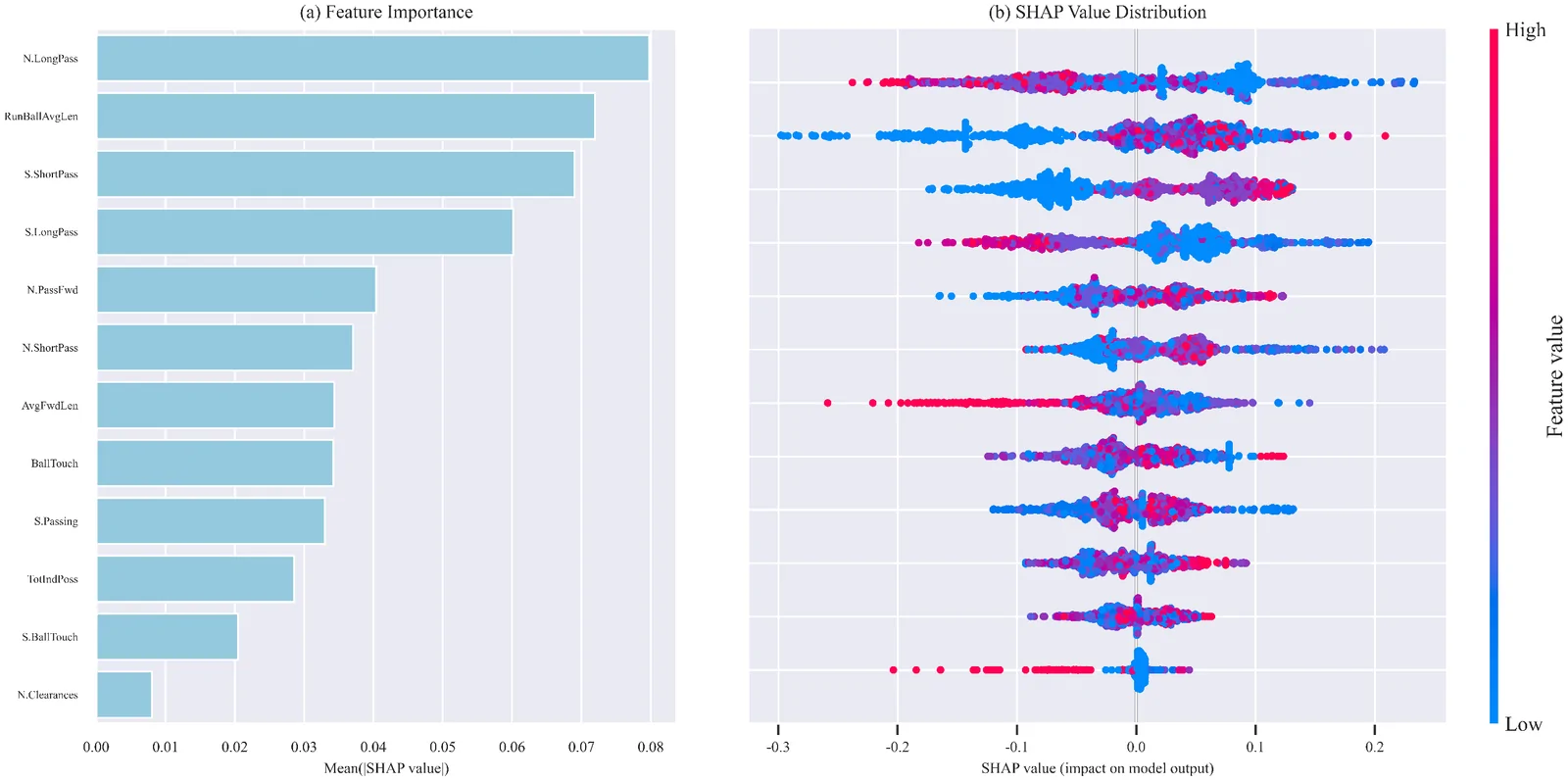
**(a)** Feature importance ranked according to the mean absolute SHAP value. **(b)** Distribution of SHAP values showing the magnitude and direction of each feature’s contribution to the model output.

**Figure 2:**
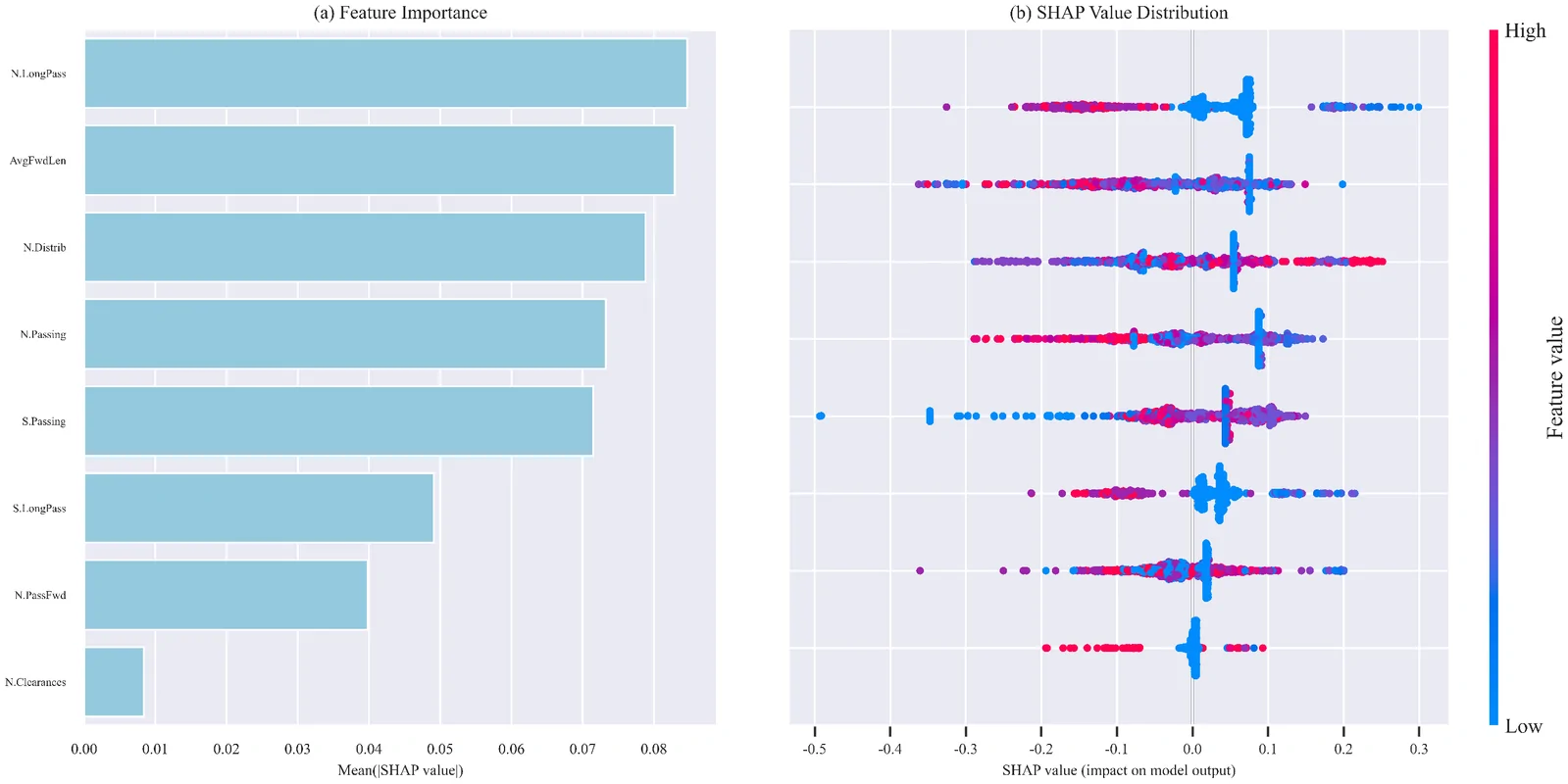
SHAP-based interpretation of technical feature contributions for classifying and predicting EDP vs. MDP (FBs).

**Figure 3:**
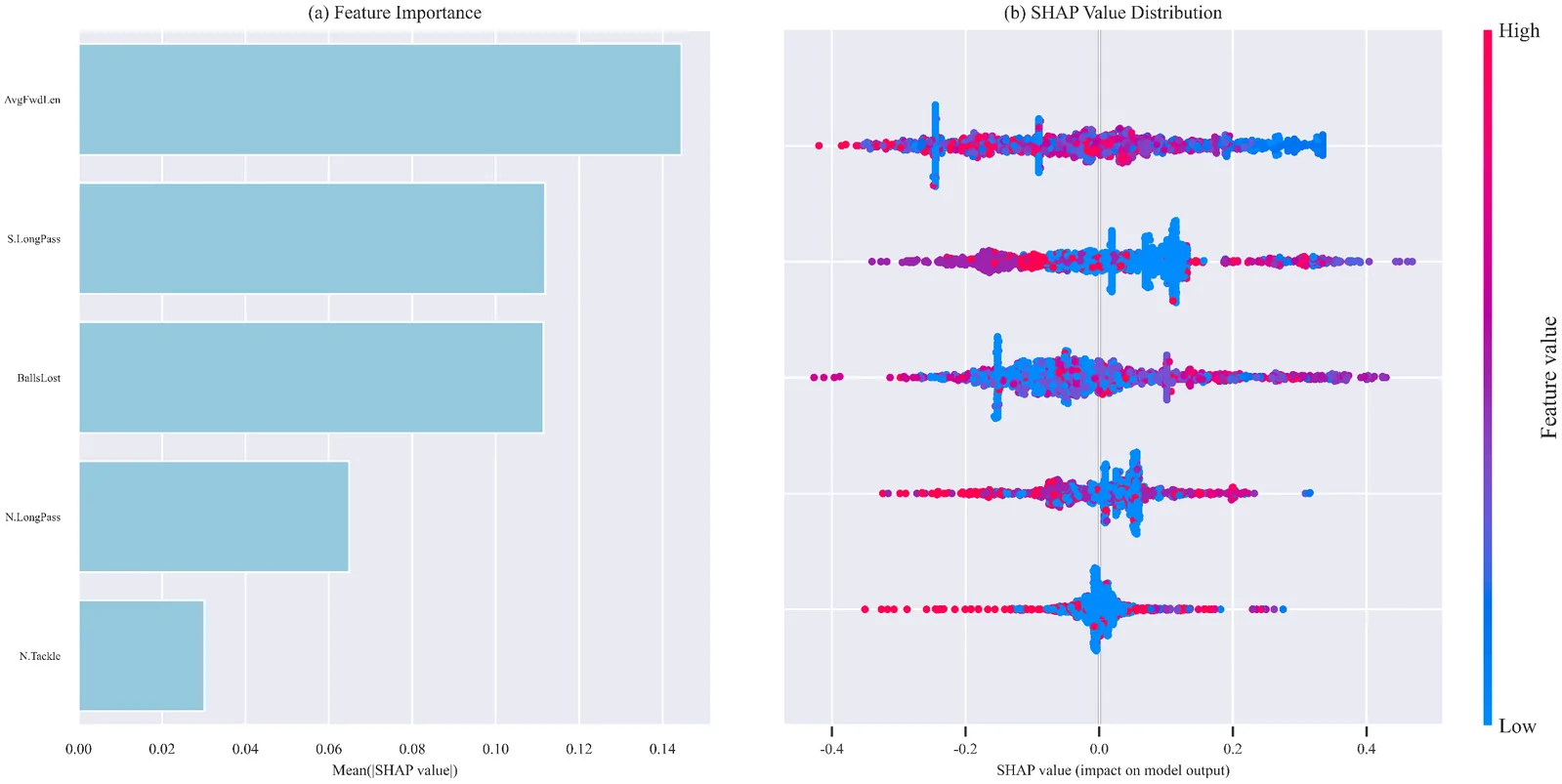
SHAP-based interpretation of technical feature contributions for classifying and predicting EDP vs. MDP (CMs).

**Figure 4:**
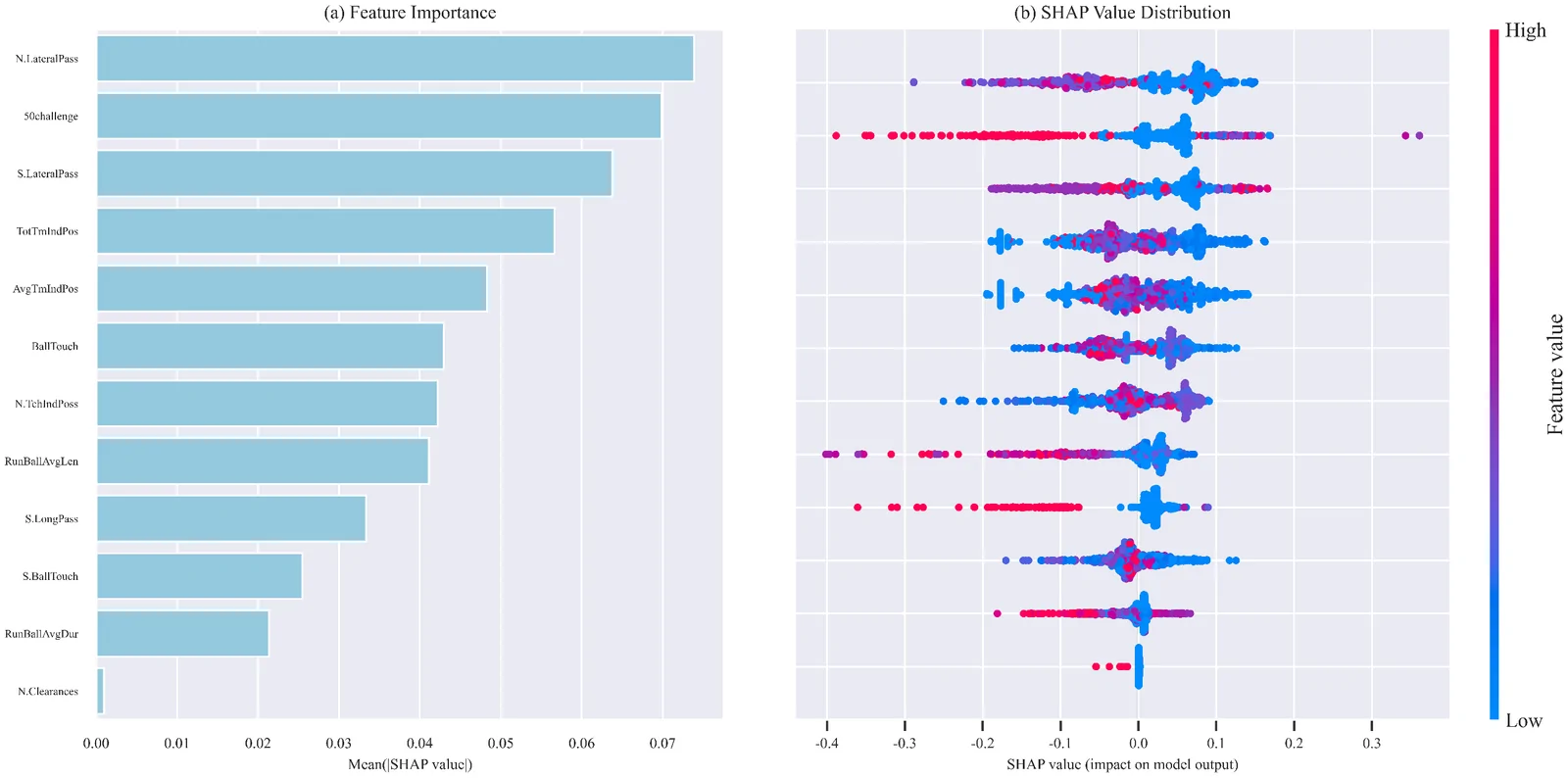
SHAP-based interpretation of technical feature contributions for classifying and predicting EDP vs. MDP (FWs).

As can be seen, For CD, significant differences were observed in variables related to passing, defensive actions, and possession, including long passes, short passes, forward passes, clearances, and individual possession. These findings were further reinforced by SHAP analysis, where features such as N.LongPass (*p* <.001; ES = -.44), S.ShortPass (*p* <.001; ES = .62), RunBallAvgLen (*p* = .01; ES = .52) and S.LongPass (*p* <.001; ES = -.42) emerged as top predictors for distinguishing EDP from MDP.

For FB, significant differences were mainly observed in long-passing activity, pass distance, and distribution frequency. These findings were further supported by SHAP analysis, where N.LongPass (*p* <.001; ES = .57), AvgFwdLen (*p* <.001; ES = .48), N.Distrib (*p* = .008; ES = .33), and N.Passing (*p* <.001; ES = .38) were the most influential predictors in distinguishing between EDP and MDP.

For CM, variables associated with defensive pressure (e.g., tackles, possession loss) and long passing showed significant differences across phases. SHAP analysis showed that features like AvgFwdLen (*p* = .006; ES = .29), S.LongPass (*p* <.001; ES = .34), BallsLost (*p* = .03; ES = .30), and N.LongPass (*p* = .02; ES = .33) are important for telling the difference between EDP and MDP.

For the WM, N.Clearances (*p* <.001; ES = .23); N.LongPass (*p* = .005; ES = .41), and Shot (*p* <.001; ES = .38) showed statistically significant differences between the EDP and MDP.

For FW, notable differences appeared in indicators of individual possession, lateral ball movement, and ball progression. The SHAP results reinforced these findings, with N.LateralPass (*p* = .02; ES = -.37), 50challenge (*p* <.001; ES = -.49), S.LateralPass (*p* = .03; ES = -.34), and TotTmIndPos (*p* = .002; ES = -.36) identified as the most influential variables for phase classification.

## 4 Discussion

The objective of this study was to examine the differences in technical performance between EDP and MDP across five outfield positions in professional football. As EDP represents the extreme end of the MDP distribution identified through position-specific thresholds, comparisons between EDP and MDP allow the examination of technical adaptations under the most demanding match conditions. The results showed a general decrease both in the quantity and quality of technical actions during EDP, with position-specific differences in player performance during extreme physical demands.

The descriptive analysis indicated that, across all playing positions, there was a general decrease in most ball control and passing-related variables during the EDP compared to the MDP, although specific factors did not universally conform to this pattern. These trends indicate that, under extreme physical stress, players are likely to engage less frequently in ball-related actions and may exhibit decreased efficiency in executing passes (Penichet-Tomas et al., 2022).

When focused specifically on variables that exhibited statistically significant differences, nearly every variable reported higher values during the MDP compared to the EDP. This suggests reduced technical involvement under EDP conditions. Rather than being explained by single physiological or cognitive mechanisms, this pattern likely reflects the combined influence of physical load, tactical constraints, and contextual match factors (Badin et al., 2016; Dambroz et al., 2022). However, the magnitude of these differences should also be considered when interpreting their practical relevance. Although several variables reached statistical significance, many effect sizes were small, indicating that these changes may represent subtle adjustments rather than substantial alterations in technical behavior. However, in elite football, where performance differences are often determined by repeated small advantages, such technical adaptations may still provide meaningful information for training design and tactical preparation.

To investigate these effects in greater detail, technical performance was evaluated individually by position. CDs demonstrated the most marked technical differences between EDP and MDP phases. Four principal indicators ranked highest in SHAP importance: number of long passes (N.LongPass: p <.001, d = 0.44), number and success of short passes (N.ShortPass, S.ShortPass: both p <.001, d > 0.60), and average running distance when in possession (RunBallAvgLen: p = .01, d = 0.52). Each of these variables reflects a distinct but complementary technical response to extreme physical demands. The reduction in N.LongPass indicates a strategic adjustment, possibly aimed at reducing turnover risk and maintaining structural compactness under conditions of fatigue (Paixão et al., 2015). On the other hand, the rise in both how often short passes are made (N.ShortPass) and how often they succeed (S.ShortPass) shows a safer, possession-focused strategy used when players might struggle with decision-making and skills due to tiredness (Lundberg and Lee, 2017). The greater average distance covered while carrying the ball shows that CDs were actively involved in advancing through individual initiative. While the main tactical actions for CDs during the MDP phase are typically associated with “covering” and “recovery runs” (Ju et al., 2022), the technical changes observed during the EDP phase suggest the adoption of more assertive and proactive behaviors to support progress (Bradley et al., 2011). The increased frequency of short passing and ball-carrying actions in this phase reinforces this positional adaptation. This discovery supports the strategic development of modern football, in which CDs serve not only as defensive anchors but also as initiators of offensive phases (Liu et al., 2016). The present findings suggest that under EDP conditions, their technical involvement becomes more diversified, with increased participation in build-up and ball progression phases. This shift in role emphasis indicates that defensive players may adapt their technical contributions depending on match intensity demands, extending beyond purely reactive behaviors.

FBs exhibited several significant technical changes between the EDP and MDP. The main influential variables included the number of long passes (N.LongPass: p <.001, ES = –0.57), average forward pass length (AvgFwdLen: p <.001, ES = 0.48), the total number of distributions (N.Distrib: p = .008, ES = 0.33), and the number and success of passes (N.Passing: p <.001, ES = 0.38; S.Passing: p = .01, ES = 0.29). These indications collectively suggest a transition toward more conservative technical behaviors under higher physical loads. The decrease in long and forward passes indicates a strategic preference toward safer options to reduce turnover risk and maintain position (Paixão et al., 2015). The decrease in overall distribution frequency may reflect reduced passing participation, possibly to minimize physical effort and save energy for key defensive responsibilities (de Pablo et al., 2024). The decrease in pass success highlights the negative impact of fatigue on technical execution, which could affect precision and decision-making in high-stress situations (Rampinini et al., 2008). Collectively, the results illustrate how FBs change their passing technique in relation to increased physical demands, prioritizing defined and low-risk choices under fatigue-related limitations.

During periods of heightened physical demand, the most prominent changes for CMs were related to passing behavior. Notably, both the number and success of long passes declined (N.LongPass: ES = 0.33; S.LongPass: ES = 0.34), alongside a reduction in the average forward pass length (AvgFwdLen: ES = 0.29). These shifts suggest a transition away from long-range distribution to more conservative ball movement, possibly as a response to increased physical pressure. This adjustment may reflect an effort to maintain control and reduce turnover risks when fatigued (Rampinini et al., 2008). Significantly, the number of ball losses increased (BallsLost: ES = 0.30), reflecting increased susceptibility to technical errors due to extreme physical and tactical pressure (Dambroz et al., 2022). This pattern indicates that CMs might turn their focus on ball recovery and defensive stability when fatigued, instead of continuing to start longer-range distributions (Kunrath et al., 2020). This adjustment may serve as a pragmatic strategy to preserve team structure during the most physically demanding phases of play, given their central positioning and frequent involvement in transitional phases (Orendurff et al., 2010; Sarmento et al., 2024).

WMs exhibited small technical differences between the EDP and the MDP. Only a limited number of variables exhibited significant changes. This small amount of change might be connected to the specific needs of the WM position, which requires constant participation in both attacking and defending, as well as a lot of quick running and sprinting (Ade et al., 2016). WMs showed relatively weak and inconsistent effects. This pattern is likely related to class imbalance, as the number of EDP observations for WMs was limited. This reduced model stability in the Random Forest analysis, resulting in their exclusion from further predictive interpretation (He and Garcia, 2009).

FWs showed noticeable declines across multiple ball control-related indicators during the EDP phase, including reductions in total ball touches (BallTouch: p = .03, ES = 0.27) and total possession time (TotTmIndPos: p = .002, ES = 0.36). These reductions indicate that FWs, who rely significantly on explosive effort, rapid decision-making, and frequent offensive participation, exhibit diminished technical performance during times of peak physical demand. These technical regressions may be attributed to positional limitations, as forward frequently operate in tight spaces and closer to defenders, which can restrict their time and space to execute technical actions under extreme pressure (Ric et al., 2017; Sarmento et al., 2024). The observed decline in lateral passes (N.LateralPass: p = .02, ES = –0.37; S.LateralPass: p = .03, ES = 0.34) may reflect limited capacity to circulate possession across horizontal channels, potentially due to restricted space or reduced teammate availability (Ric et al., 2017). Additionally, the decrease in 50 challenges (50challenge: p <.001, ES = 0.49) suggests lower engagement in direct physical contests, possibly stemming from fatigue-induced reductions in explosive strength (Thorlund et al., 2009).

To complement the univariate statistical analysis, the Random Forest and SHAP framework provided a multivariate perspective on the relative importance of technical variables in distinguishing between EDP and MDP conditions. Across playing positions, the SHAP results indicated that changes in technical behavior during EDP were mainly driven by passing and ball progression actions. In particular, short-passing frequency, long-passing involvement, and ball-carrying activity consistently contributed most to the observed differences between EDP and MDP conditions. These patterns suggest that technical adjustments under EDPs are not random or isolated. Instead, they reflect systematic changes in how teams and players manage ball circulation under pressure. For example, increased reliance on short passes indicates a shift toward maintaining possession stability, while changes in long-pass frequency reflect reduced willingness to attempt riskier forward distribution under high physical load. Ball-carrying actions also emerged as a key factor, indicating a redistribution of technical behaviors toward individual progression under EDP conditions. This indicates that players may adapt their technical behavior by balancing risk and control depending on physical demand intensity. Overall, these findings highlight that the differences between EDP and MDP are primarily expressed through coordinated adjustments in passing strategy and ball progression rather than isolated technical actions.

### 4.1 Limitation

This study has several limitations that should be acknowledged. First, the use of a fixed high-intensity running (HIR) threshold (≥19.8 km·h^-^¹) and 5-minute rolling windows, although consistent with previous studies, may not fully capture very short and intermittent bursts of high-intensity activity. Such brief fluctuations are increasingly relevant in modern match play and may therefore be underestimated by the current analytical approach (Oliva-Lozano et al., 2020). Second, contextual match-related factors, including match status, opponent quality, and tactical strategies, were not included in the analysis. These variables are known to influence both physical output and technical performance, and their absence may limit the contextual interpretation of EDP-related differences (Oliva-Lozano et al., 2023). Third, while several results reached statistical significance, many effect sizes were small. This indicates that the practical relevance of some observed differences may be limited, and findings should therefore be interpreted cautiously when applied to real-world coaching or performance settings. Fourth, the relatively weak predictive performance observed for wide midfielders may be attributed to data imbalance, as the number of EDP observations in this position was relatively low. This imbalance likely reduced the stability of positional comparisons and may have affected the robustness of the observed patterns for this role. Finally, the present study focused exclusively on elite professional football, which may limit the generalizability of the findings to other competitive levels such as youth or semi-professional players, where physical and technical demands may differ substantially. Future research should consider more refined temporal approaches for identifying peak demands and incorporate broader contextual variables to improve the ecological interpretation of EDP-related performance patterns across different populations and playing styles.

### 4.2 Practical applications

This study’s findings provide valuable information for practitioners aiming to improve technical performance during periods of heightened physical load. Although several differences between EDP and MDP conditions showed small effect sizes, these changes may still provide meaningful information for practitioners because repeated small adaptations can influence tactical execution during high-pressure situations. Considering that EDP phases inflict significantly higher physiological stress than MDP phases, training approaches should replicate high-intensity match situations to improve players’ technical stability and decision-making under fatigue. This can be achieved through specific training designs such as small-sided games (SSGs) with manipulated constraints, including numerical disadvantages (e.g., 3v4 or 4v5), reduced pitch dimensions, and intermittent high-intensity bouts to simulate EDP conditions. For FWs, SSGs with numerical disadvantages and limited space can be used to enhance decision-making speed and ball control under pressure during peak exertion. FBs may benefit from wide-area possession games emphasizing rapid short-passing sequences under overload conditions and repeated sprint transitions. For CDs, directional possession games with progressive build-up tasks can be applied to improve short passing and ball progression under fatigue. CMs necessitate transition-based SSGs focusing on defensive pressure resistance and error reduction in congested central areas. Given that WMs showed relative stability, training may priorities consistency maintenance through balanced SSG formats while supporting multi-role involvement. Position-specific adaptations should be integrated into tactical planning, workload management, and recovery protocols to maintain performance during periods of heightened physical load.

## 5 Conclusions

This study analyses the technical performance differences between EDP and MDP across five outfield positions. The results revealed a general decline in both the volume and effectiveness of technical actions under higher physical loads, with notable variation across playing positions. FWs exhibited the most pronounced deterioration, particularly in ball control and dueling actions. FBs demonstrated conservative adaptations in the passing strategy, reducing long and forward pass length. CDs responded with increased short passing and ball-carrying distances, while CMs showed more frequent defensive actions and greater susceptibility to ball loss. In contrast, WMs experienced minimal changes across the two phases. These findings underscore the differentiated impact of extreme physical demands on technical execution across positions and suggest that tactical planning and training strategies should be tailored to account for these positional nuances.

## Data Availability

The datasets presented in this article are not readily available because the study involved the analysis of anonymized football performance data collected from official matches organized by the CFA. The CFA provided consent for the use of these data for research purposes. Given the retrospective and anonymized nature of the data, the Ethics Committee approved the waiver of written informed consent from participants. Requests to access the datasets should be directed to nzq10@hotmail.com.
